# The Cytotoxic Effects of Betulin-Conjugated Gold Nanoparticles as Stable Formulations in Normal and Melanoma Cells

**DOI:** 10.3389/fphar.2018.00429

**Published:** 2018-05-03

**Authors:** Marius Mioc, Ioana Zinuca Pavel, Roxana Ghiulai, Dorina E. Coricovac, Claudia Farcaş, Ciprian-Valentin Mihali, Camelia Oprean, Vlad Serafim, Ramona A. Popovici, Cristina A. Dehelean, Michael I. Shtilman, Aristidis M. Tsatsakis, Codruţa Şoica

**Affiliations:** ^1^Faculty of Pharmacy, Victor Babeş University of Medicine and Pharmacy, Timişoara, Romania; ^2^“George Emil Palade” Electron Microscopy Center, Institute of Life Sciences, “Vasile Goldiş” Western University of Arad, Arad, Romania; ^3^“Pius Brînzeu” Timişoara County Emergency Clinical Hospital, Oncogen Institute, Timişoara, Romania; ^4^Department of Natural Sciences, Middlesex University London, London, United Kingdom; ^5^Faculty of Dentistry, Victor Babeş University of Medicine and Pharmacy, Timişoara, Romania; ^6^Dmitry Mendeleev University of Chemical Technology of Russia, Moscow, Russia; ^7^Department of Forensic Sciences and Toxicology, Faculty of Medicine, University of Crete, Heraklion, Greece

**Keywords:** gold nanoparticles, betulin, skin, apoptosis, melanoma

## Abstract

Gold nanoparticles are currently investigated as theranostics tools in cancer therapy due to their proper biocompatibility and increased efficacy related to the ease to customize the surface properties and to conjugate other molecules. Betulin, [lup-20(29)-ene-3β, 28-diol], is a pentacyclic triterpene that has raised scientific interest due to its antiproliferative effect on several cancer types. Herein we described the synthesis of surface modified betulin-conjugated gold nanoparticles using a slightly modified Turkevich method. Transmission electron microscopy (TEM) imaging, dynamic light scattering (DLS), scanning electron microscopy (SEM) and energy-dispersive X-ray spectroscopy (EDX) were used for the characterization of obtained gold nanoparticles. Cytotoxic activity and apoptosis assessment were carried out using the MTT and Annexin V/PI apoptosis assays. The *in vitro* results showed that betulin coated gold nanoparticles presented a dose-dependent cytotoxic effect and induced apoptosis in all tested cell lines.

## Introduction

Metallic nanoparticles are currently investigated as drug delivery systems and/or diagnostic agents in cancer therapy due to their unique physico-chemical properties and their ability to improve drug selectivity against cancer cells (Ahmad et al., 2010). Gold nanoparticles were described as biocompatible and highly effective when compared to other metallic nanoparticles applied in antitumor therapy as drug nanocarriers or hyperthermia agents (Khan et al., 2014). Basically, gold nanoparticles provide multiple assets for biomedical applications, as: controlled synthesis, easy functionalization, passive/active targeting properties and the ability to transport and protect large amounts of loaded drugs (Ahmad et al., 2010). The use of gold nanoparticles as drug delivery platforms can be accomplished via conjugating the active drugs with gold nanoparticles usually by the use of spacers such as thiolated polyethylene glycols that covalently bind to gold; the final goal being the achievement of plasmatic stability and selectivity, as well as the ability to release the active drug as a result of metabolic processes (Cui et al., 2017). Another approach is the direct deposition of the active compound on the surface of gold nanoparticles (Elbialy et al., 2014), probably for the fact that gold nanoparticles might be considered as centers of formation of a new phase due to adsorption of precipitated substances. Several anticancer drugs such as doxorubicin (Jang et al., 2013), recombinant human endostatin (Li et al., 2016) and paclitaxel (Heo et al., 2012; Paciotti et al., 2016) were loaded in gold nanoparticle-based delivery systems, thus achieving significantly improved nuclear uptake, targeted release and an overall increased antitumor efficacy. In fact, a certain level of selectivity provided by the enhanced permeability and retention effect, which occurs for all nanosized drugs, is caused by the leaky vasculature within tumor microenvironment (Nakamura et al., 2016). The biocompability and tunable optical and electronic properties make gold nanoparticles suitable agents in the biomedical field (Gao, 2013; Piperigkou et al., 2016; Engin et al., 2017). In particular, there are a few characteristics such as small size, inert nature and the opprtunity to bind to multiple surface ligands which allow gold nanoparticles to penetrate inside cells and deliver their payloads without eliciting significant immune response (Garrido et al., 2015). As a result, several gold nanoparticle based formulations have reached preclinical or clinical trials as theranostic agents in cancer therapy (Pedrosa et al., 2015).

Betulin, [lup-20(29)-ene-3β,28-diol], is a pentacyclic triterpene that has drawn scientific interest due to its multiple biologic activities, mainly anti-inflammatory and antitumor (Alakurtti et al., 2006); in spite of this promising pharmacological profile, its clinical use is severely restricted by its very poor bioavailability and bio-distribution in direct correlation to its low water solubility (Drąg-Zalesińska et al., 2017). In an effort to overcome this drawback, several nanoformulations with betulin have been developed such as cyclodextrin complexes (Soica C. et al., 2012), polymer-liposome nano-complexes (Cho et al., 2007), nanoemulsions (Dehelean et al., 2013), carbon nanotubes (Saudagar and Dubey, 2014), nanostructured carbon sorbent (Lavrenov et al., 2015); all studies reported an improved pharmacokinetic profile of the active compound therefore an increased bioavailability.

In the current study, we report the preparation of betulin-conjugated gold nanoparticles by using sodium citrate as both reducing agent and stabilizer; two types of conjugated nanoparticles were synthesized, with and without thiolated polyethylene glycol, respectively. The physico-chemical parameters of the final nanoparticles were assessed by employing various analytical techniques due to the fact that nanoparticles’ size, shape and stability hugely influence their future biological behavior (Dreaden et al., 2012). The *in vitro* biological activity of betulin-conjugated gold nanoparticles was further analyzed by means of MTT and Annexin V/PI assays.

## Materials and Methods

### Materials

Betulin, nitric acid, hydrochloric acid and L-Cysteine hydrochloride were purchased from Fisher Scientific (Loughborough, United Kingdom). Chloroauric acid (HAuCl_4_), trisodium citrate dihydrate (C_6_H_5_O_7_Na_3_⋅2H_2_O), and poly(ethylene glycol) 2-mercaptoethyl ether acetic acid (HOOC-Peg-SH, MW 3500) were purchased from Sigma-Aldrich (Taufkirchen, Germany) and used as received. The deionized water was prepared by using the Ultrapure, Millipore^®^ Direct-Q^®^ 3 with UV radiation.

### Gold Nanoparticles Synthesis

The synthesis of gold nanoparticles was conducted according to the previously published Turkevich procedure with slight modifications (Sutherland and Winefordner, 1992; Kim et al., 2007). Briefly, 380 ml deionized water were heated to boiling under vigorous stirring; HAuCl_4_ solution (a volume of 1.44 ml – 4% concentration) was added followed by 18.6 ml of aqueous solution containing 175 mg of trisodium citrate dihydrate (C_6_H_5_O_7_Na_3_ ⋅2H_2_O) while maintaining the boiling temperature for approximately 10–15 min. The molar ratio was 1:3.5 (HAuCl_4_: C_6_H_5_O_7_Na_3_ ⋅2H_2_O). The transparent, ruby-red solution was allowed to cool at room temperature. Purification was accomplished by centrifugation (30 min at 15000 rpm) followed by repeated washing with deionized water. The final product was lyophilized and resuspended in methanol for further investigation. PEG-surface modified gold nanoparticles were prepared by adding 10 mg of HOOC-PEG-SH to 100 ml of aqueous gold nanoparticle solution (Alkilany et al., 2015), under continuous stirring for about 30 min. The resulting solution was centrifuged for 30 min at 15000 rpm and the final product was lyophilized and resuspended in the required solvent.

### Betulin Loading Onto Gold Nanoparticles

Betulin (Bet) loading was accomplished by adding, under continuous stirring, 2 ml of betulin solution in methanol (1 mg/ml) to 2 ml colloidal gold solution in methanol, achieving a relative betulin:gold molar ratio (gold molarity related to chloroauric acid used for the reduction) of 1:4; methanol was selected as a dispersion medium for both AuNP and betulin in order to avoid betulin precipitation. The dispersion medium was subsequently removed by vacuum evaporation; the resulting powder was resuspended in the desired solvent.

### UV-Vis Spectroscopy

The UV-Vis analysis was performed using a UV-VIS spectrophotometer, series T80 (PG Instruments LtD, United Kingdom), with a quartz cuvette at 25°C temperature in the wavelength range of 400–750 nm. In order to evaluate the gold nanoparticles formation process, spectrophotometric measurements were achieved regularly (each minute) throughout the synthesis. The stability of the colloidal gold solution was evaluated by spectrophotometry daily for 7 days, the samples being stored in the refrigerator (4°C) throughout the experiment.

### FTIR Spectroscopy

The Fourier-transform Infrared Spectroscopy (FTIR) spectra were recorded using a Shimadzu Prestige-21 spectrometer; the samples were processed as potassium bromide pellets within the range of 400–4000 cm^-1^ at 4 cm^-1^ resolution.

### Quantification of Gold From Gold Nanoparticles Using Inductively Coupled Plasma – Mass Spectrometry (ICP – MS)

#### Sample Preparation

A volume of 1 mL mixture of nitric acid and hydrochloric acid (25:75, %v/v) containing 600 ppb of internal standard (Iridium) was added to 20 μL of gold nanoparticles suspension. The mixture was incubated in a fume hood overnight, at room temperature. After incubation, the suspension obtained was further diluted in a mixture of 1% hydrochloric acid and 1% L-Cysteine at a 1:60 ratio. The final dilution of the initial sample was 1:300.

#### Gold Determination

Analysis was performed using a XSeries 2 ICP-MS coupled to a Cetac ASX-520 autosampler (Thermo Fisher Scientific, United States). The acquisition mode consisted in peak jumping with two channels, one mass per channel: 193Ir (Iridium) and 106Au (Gold). Acquisition parameters were: dwell time – 100 ms for 106Au and 50 ms for 193Ir, separation AMU – 0.02, number of sweeps – 50, the acquisition time – 7 s. Iridium was set as internal standard and gold for quantitation. RF power was set at 1400 W and nebulizer set concentric at 35 psi with 1 mL/min flow. Sample uptake delay was set with a minimum of 40 s and a maximum of 50, while the washout maximum delay was set at 65 s. The washing solution used was 1% hydrochloric acid and 0.1% L-Cysteine. The blank sample consisted of 1% hydrochloric acid, 1% L-Cysteine and 10 ppb Iridium. The limit of detection (LOD) was calculated using 3.14σ of 7 consecutive blank samples and the limit of quantification was calculated using 10σ of 7 consecutive blank samples. For calibration, the curve fitting options were: line fit – linear, weight – none, and force – through blank.

### Particle Size and Zeta Potential Analysis

In order to determine the size and stability of the aqueous solutions for the studied samples, Vasco Particle Size Analyzer and Wallis Zeta-potential Analyzer (Cordouan Technologies, France) were utilized. For data acquisition, the following parameters for the Size Analyzer were preset: temperature 25°C, time interval 14 μs, number of channels 337, laser power 95%, DTC position down, acquisition mode continuous, analysis mode Cumulants and for the Zeta potential Analyzer: temperature 25°C, plastic cuvette, medium resolution, laser power 75%, electrode distance 5 mm, Henry function Huckel.

### TEM, SEM-EDAX Analysis

The morphology of the sample was characterized by transmission electron microscopy (TEM) using a FEI Tecnai 12 Biotwin microscope.

SEM-EDAX analysis was carried out using an EDAX detector (ZAF Quantification – Standardless, Element Normalized) with FEI Quanta 250 microscope. SEM analysis parameters were: HV mode, 30 kV, ETD (Everhart-Thornley detector for secondary electrons), two magnification orders, one for a general overview image/measurements and another one for higher surface topography sides analysis. The identified chemical species were expressed in weight percent (Wt %) or atomic percent (At %).

### Cell Culture

Human melanoma cell line – A375 (ATCC^®^ CRL-1619^TM^) was purchased from the American Type Culture Collection (ATCC, Manassas, VA, United States); B164A5 (Sigma Aldrich 94042254) murine melanoma cell line was acquired from Sigma-Aldrich (Taufkirchen, Germany); 1BR3 human skin fibroblast (ECACC 90011801) was obtained from the European Collection of Authenticated Cell Cultures (ECACC, Salisbury, United Kingdom); the HaCaT (human keratinocyte) cells were kindly provided by the University of Debrecen, Hungary.

A375, B164A5 and HaCaT cells were cultured as a monolayer in high glucose Dulbecco’s Modified Eagle’s Medium (DMEM; Sigma-Aldrich, Taufkirchen, Germany) supplemented with 10% fetal bovine serum (FBS; Gibco, ThermoFisher Scientific) and 1% Penicillin/Streptomycin mixture (Pen/Strep, 10,000 IU/ml; Sigma-Aldrich, Taufkirchen, Germany). 1BR3 cells were grown in Eagle’s Minimum Essential Medium (EMEM; ATCC) supplemented with 15% fetal bovine serum (FBS; Gibco, ThermoFisher Scientific) and 1% Penicillin/Streptomycin mixture (Pen/Strep, 10,000 IU/ml; Sigma-Aldrich, Taufkirchen, Germany). The cells were kept in standard conditions (humidified atmosphere containing 5% CO_2_, 37°C).

### Cytotoxicity Assay

The *in vitro* cytotoxicity was determined by means of 3-(4,5-dimethylthiazol-2-yl)-2,5-diphenyltetrazolium bromide (MTT) method. In brief, HaCaT, 1BR3, A375 and B164A5 cells were seeded in 96-well plates at an initial density of 1 × 10^4^ cells/well and allowed to attach. Next, the old medium was removed and a fresh one was added containing AuNP formulations in dimethyl sulfoxide – DMSO (AuNP, AuNP + Bet, AuNP Peg, and AuNP Peg + Bet) at the final concentration of 10 and 50 μM equivalent of betulin, and the cells were incubated for 24, 48, and 72 h. The control cells were treated with the same amount of DMSO, the highest concentration of DMSO present in the medium being 0.5%. A volume of 10 μL MTT reagent (5 mg/mL) was added in each well. During a 4 h contact period, the intact mitochondrial reductase converted and precipitated MTT as blue crystals. The precipitated crystals were dissolved in 100 μL of lysis solution provided by the manufacturer (Sigma-Aldrich). Finally, the reduced MTT was spectrophotometrically analyzed at 570 nm, using a microplate reader (xMark Microplate Spectrophotometer, Bio-Rad). The *in vitro* experiments were carried out in triplicate.

### Annexin V/PI Apoptosis Assay

For the flow cytometric analysis of cell apoptosis, an Annexin V-FITC kit (Invitrogen, ThermoFisher, Vienna, Austria) was used. A number of 5 × 10^5^cells/well were seeded onto 6-well plates (Greiner bio-one) and incubated with the tested compounds for 72 h. The selected concentrations were 10 and 50 μM, which were obtained by successive dilutions into the culture medium starting from a stock solution of 10 mM in DMSO. Untreated cells were used as control; cells treated with DMSO were used as solvent control. The highest DMSO concentration (0.5%) in the medium did not exhibit any significant effect on cell apoptosis. After trypsinization, cells were collected, resuspended in 500 μL Phosphate Buffer Saline (Sigma-Aldrich) and centrifuged for 5 min at 1500 RPM. The next step consisted in performing the flow cytometric studies according to the manufacturer’s protocol: 2–5 × 10^5^cells were two times washed in 1 × Annexin V Binding Buffer, centrifuged at 1500 RPM for 5 min, resuspended in the binding buffer and incubated with 5 μL of Annexin V-FITC for 15 min in the dark. After washing the cells with 200 μL specific binding buffer and centrifugation, the cells’ pellet was resuspended in 190 μL binding buffer, and 10 μL of PI solution was added immediately prior to analysis by flow cytometry. The data were achieved using a FACSCalibur flow cytometer (Becton Dickinson, Franklin Lakes, NJ, United States). The results were analyzed using Flowing Software 2.5.1.

## Results

### UV-Vis Spectrophotometry

**FIGURE 1:**
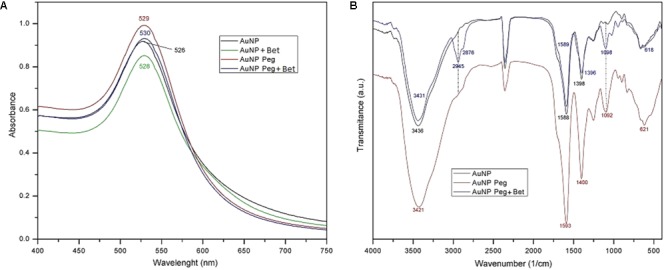
Recorded UV-Vis spectra for AuNP formulations: AuNP, AuNP + Bet, AuNP Peg and AuNP Peg + Bet **(A)** and FTIR spectra of AuNP formulations **(B)**.

Along synthesis, UV-Vis spectrophotometry revealed the shift of the absorption maximum toward larger wavelengths. The formation of gold nanoparticles was monitored in terms of color change and the UV-Vis analysis generated a single absorption peak for each type of gold compounds that in the end of the synthesis were located in the interval of 520–530 nm, thus indicating the formation of gold nanoparticles and justifying the ruby-red color of the final solution (**Figure 1A**). The corresponding wavelengths for maximum peak absorption were: 526 nm for AuNP, 528 nm for AuNP + Bet, 529 nm for AuNP PEG and 530 nm for AuNP PEG + Bet (**Figure 1A**). Also, stability studies were conducted at 7 days, 1 and 3 months, respectively, after the nanoparticle synthesis, by employing UV-Vis spectrophotometry; the absorption maximum remains situated around 520 nm thus indicating insignificant changes of the AuNP dimensions.

### FTIR Spectroscopy

FTIR analysis revealed the characteristic bands of the various AuNP specimens (**Figure 1B**). The vibrational spectra were recorded in the range of 400–4000 cm^-1^. The band located at 3421/3431/3436 cm^-1^ identified in all the studied samples can be assigned to the O-H stretching vibration (Schrader, 1995), hydroxyl bonds being present in both citrate and betulin. The band located at 2945 cm^-1^ attributed to the =C-H stretch and the one at 2876 cm^-1^ assigned to the C-H stretch, indicate the presence of betulin (Joshi et al., 2013; Wang et al., 2014) in the sample AuNP Peg Bet; these bands were not present in the other two samples that lack betulin. The other bands assigned to betulin, in the literature, are overlapped with the ones attributed to the other components present in the sample.

The intense absorption peaks located around 1590 and 1395 cm^-1^ belong to the symmetric and asymmetric stretching vibration of COO^-^ in trisodium citrate (Bai et al., 2010; Mohan et al., 2013). Due to the fact that these bands are shifted as compared to the ones reported for the pure trisodium citrate (Bai et al., 2010), one can assume the conjugation of the carboxyl groups at the surface of gold nanoparticle. In addition, another typical band located around 620 cm^-1^ indicate the presence of citrate alkyl groups. The band located at 1092/1098 cm^-1^ is characteristic for the C-O-C stretching vibration (Vonnemann et al., 2014), certifying the presence and functionalization of PEG-SH.

### Gold Determination by ICP-MS

The gold LOD (limit of detection) was calculated at 0.0035 μg/L and the LOQ at 0.0111 μg/L. Calibration correlation coefficient was 0.9998. The dilution factor was applied to the obtained results. The quantitative results are displayed in **Table 1**.

**Table 1:** Gold concentration of nanogold samples determined by ICP-MS.

Sample	Concentration (μg Au/mL suspension)
AuNP	30.2511
AuNP Peg	35.3736
AuNP + Bet	28.4274
AuNP Peg + Bet	28.6324

### Particle Size and Zeta Potential Analysis

The results of the particle size and zeta potential are presented in **Table 2**. The Cumulants analysis provided by the Particle Size Analyzer offers an average size of the particles in the analyzed sample. One can notice a slight dimension growing tendency from AuNP to AuNP + Bet and AuNP Peg-SH that can be explained by the addition of Bet and Peg-SH, respectively in the sample. AuNP Peg-SH + Bet show the largest dimensions (88 ± 10 nm).

**Table 2:** Particle size and zeta-potential values of the nanogold samples.

Sample	Particle size (nm)		Zeta-potential (mV)
	Cumulants		
	Mean ± *SD*	Intensity	
AuNP	43 ± 5	0.8	–52.87
AuNP + Bet	66 ± 7	0.7	–46.19
AuNP Peg	67 ± 8	0.8	–27.59
AuNP Peg + Bet	88 ± 10	0.8	–34.76

*SD, standard deviation.*

### TEM, SEM-EDX Analysis

**FIGURE 2:**
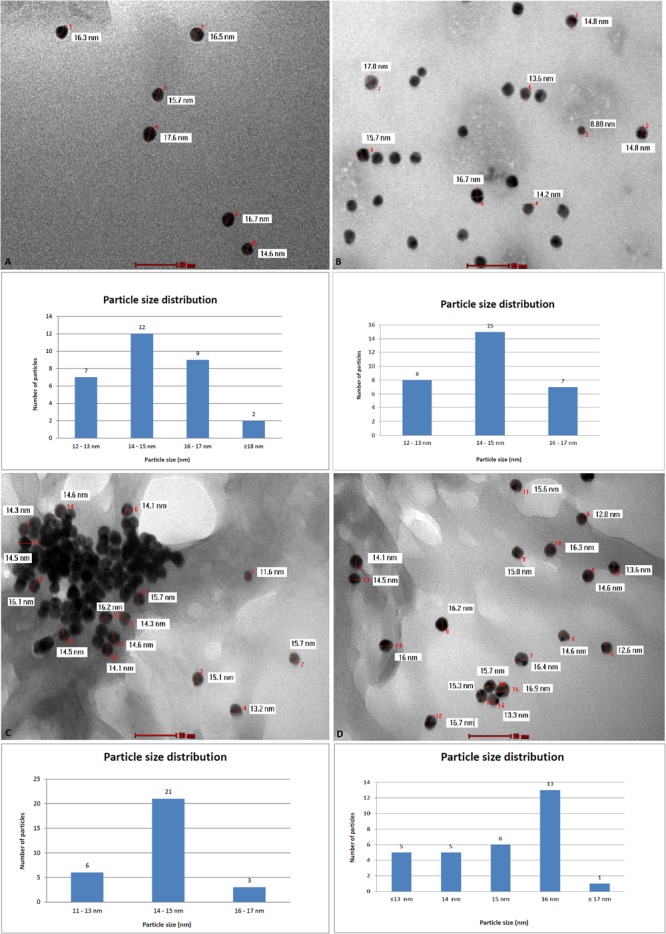
Transmission electron microscopy (TEM) analysis and particle size distribution for nanogold samples: AuNP **(A)**, AuNP Peg **(B)**, AuNP + Bet **(C)**, AuNP Peg + Bet **(D).**

TEM analysis revealed an average particle diameter situated around 14–15 nm for all samples (**Figure 2**). In addition, the analysis highlighted the spherical shape of the gold nanoparticles regardless of their composition.

**FIGURE 3:**
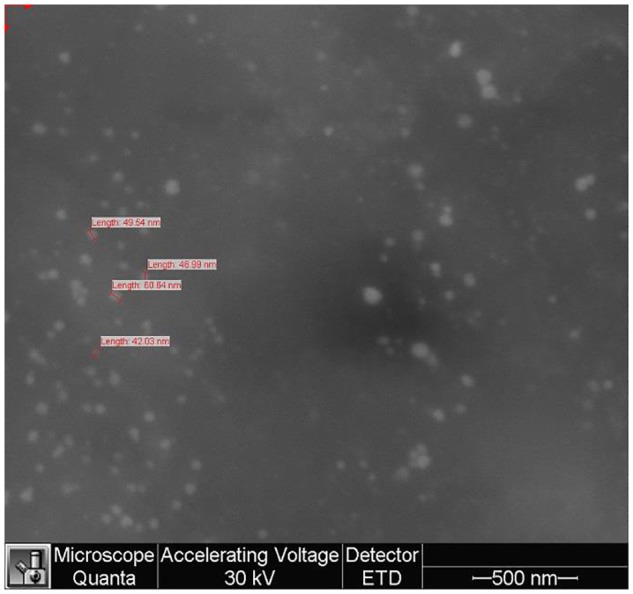
Scanning electron microscopy (SEM) analysis of gold nanoparticles sample (AuNP).

**Figure 3** shows the SEM image of the sample AuNP; one can notice spherical shapes of various sizes (42.03–60.64 nm) with contact facets among them and also an agglomeration tendency.

**FIGURE 4:**
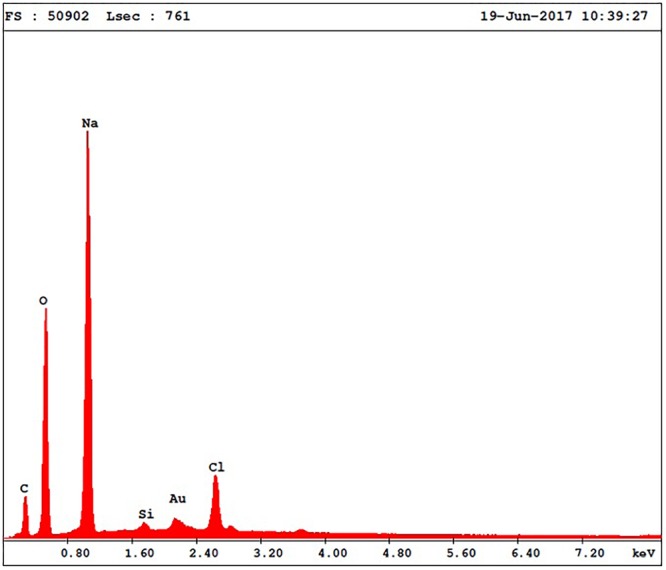
EDX spectra of gold nanoparticles sample (AuNP).

The energy dispersive X-ray analysis (EDX) (**Figure 4**) was employed to determine the elemental composition of the sample (**Table 3**); the analysis showed the presence of C, O, Na, Au, and Cl in the samples thus confirming nanoparticle formation.

**Table 3:** Elemental composition of the Au-NPs.

Element	Wt%	At%	*m+K*-Ratio	*Z*	*A*	*F*
C K	21.86	31.27	0.0469	1.0384	0.2066	1.0005
O K	38.14	40.96	0.0962	1.0238	0.2460	1.0008
NaK	34.85	26.04	0.0920	0.9618	0.2745	1.0002
AuM	2.70	0.54	0.0193	0.7834	1.1567	1.0002
ClK	2.45	1.19	0.0152	0.9409	0.6594	1.0000
Total	100.00	100.00				

### Cytotoxicity Assay

**FIGURE 5:**
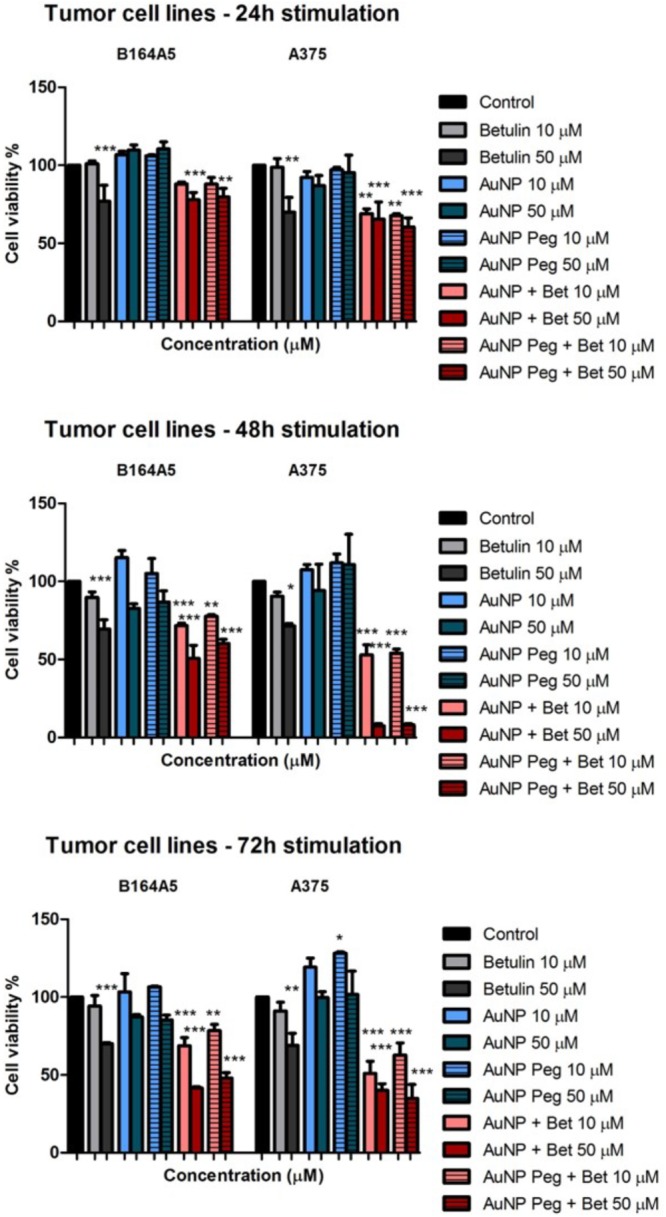
*In vitro* cytotoxicity assessment of naked AuNPs/AuNP Peg and betulin conjugated gold nanoparticles (10 and 50 μM) on tumor cells: murine – B164A5 and human – A375 melanoma cells at 24, 48, and 72 h post-stimulation by the means of MTT assay. The results are expressed as cell viability percentage (%) related to control cells (stimulated with dimethyl sulfoxide – DMSO). The data represent the mean values ± SD of three independent experiments performed in triplicate. One-way ANOVA analysis was applied to determine the statistical differences followed by Tukey post-test (^∗^*p* < 0.05; ^∗∗^*p* < 0.01; ^∗∗∗^*p* < 0.001).

The percentage of viable cells after incubation with test compounds was calculated related to the solvent control (DMSO used for samples preparation). Cell viability results obtained on tumor cell lines are depicted in **Figure 5**.

At 24 h post-stimulation AuNP and AuNP Peg induced a slightly increase in B164A5 murine melanoma cells viability, whereas on A375 elicited a modest decrease in cell viability. AuNPs loaded with betulin induced a decrease in cell viability in a dose-dependent manner, the decrease being more potent in the case of A375 human melanoma cells (**Figure 5**). The cytotoxic effect induced by both AuNPs and AuNP Peg loaded with betulin was slightly higher as compared with the one recorded for betulin alone at the same concentration. At 48 h post-stimulation, AuNP and AuNP Peg did not significantly affect cancer cell viability. A small decrease of cell viability was noticed only in the case of B164A5 cells, at the highest tested concentration (50 μM). Betulin-conjugated AuNPs and AuNP Peg proved to be significantly toxic mainly for human melanoma cells – A375 when compared with betulin alone at 48 h post-stimulation. Similar effects were noticed in the case of murine melanoma cells – B164A5, showing that betulin-conjugated gold nanoparticles exhibit a stronger *in vitro* cytotoxic effect compared to betulin. Even after 72h incubation, AuNP and AuNP Peg lacked any cytotoxic activity against A375 cells, moreover a slightly stimulatory effect was recorded; for B164A5 cells a decrease in cell viability was reported when the highest concentration (50 μM) was used. However, on both types of melanoma cells, betulin-conjugated gold nanoparticles elicited a decrease in cell viability in a dose-dependent manner; their cytotoxicity was significantly higher related to betulin as such (**Figure 5**).

**FIGURE 6:**
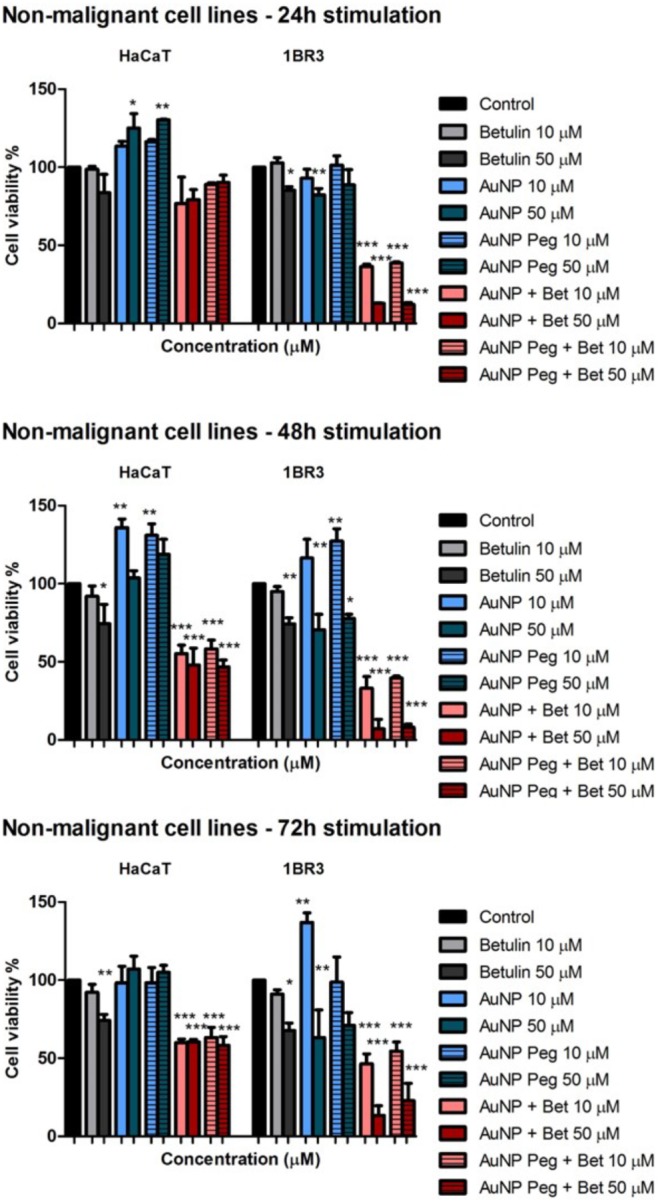
*In vitro* cytotoxicity assessment of naked AuNPs/AuNP Peg and betulin conjugated gold nanoparticles (10 and 50 μM) on non-malignant human cells: keratinocytes – HaCat and fibroblasts – 1BR3 melanoma cells at 24, 48, and 72 h post-stimulation by the means of MTT assay. The results are expressed as cell viability percentage (%) related to control cells (stimulated with dimethyl sulfoxide – DMSO). The data represent the mean values ± SD of three independent experiments performed in triplicate. One-way ANOVA analysis was applied to determine the statistical differences followed by Tukey post-test (^∗^*p* < 0.05; ^∗∗^*p* < 0.01; ^∗∗∗^*p* < 0.001).

All nanogold formulations were also assessed for their cytotoxicity against two non-malignant cell lines (HaCaT, 1BR3). The results are presented in **Figure 6**. HaCaT cells stimulation with AuNP and AuNP Peg led to increased cell viability at all three evaluated time points (24, 48, and 72 h); the 1BR3 cells proved to be more sensitive in a dose- and time-dependent manner, the lowest viability percentage being recorded for the highest concentration (50 μM) after 72 h incubation. Treatment with betulin-conjugated gold nanoparticles reduced cell viability, in particular for the 1BR3 cell line (skin fibroblasts) in a dose-dependent manner, the highest cytotoxic effect being recorded at 48 h. Betulin alone proved to be less toxic on both human keratinocytes – HaCat and fibroblasts – 1BR3 cell lines by comparison with betulin loaded gold nanoparticles. Seventy two hour stimulation with AuNP + Bet and AuNP Peg + Bet induced a significant decrease in 1BR3 cell viability, in particular at the highest tested concentration (50 μM), whereas the keratinocytes exhibited higher viability rates.

### Annexin V/PI Apoptosis Assay

**FIGURE 7:**
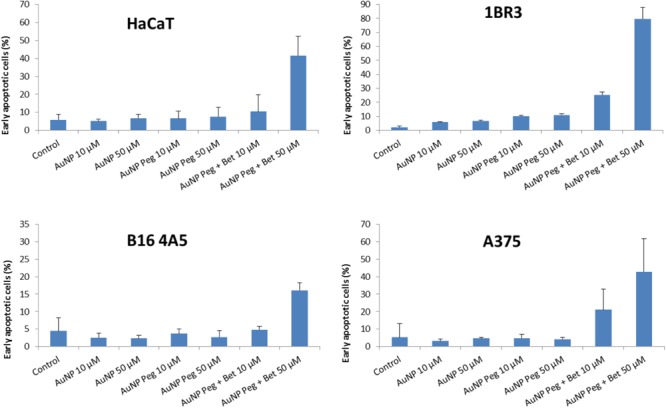
Summarized results of early apoptotic cells percentage of HaCaT, 1BR3, B16 4A5 and A375 cell lines after 72 h of treatment with the tested substances. The results are representative of three independent experiments in triplicate and expressed as mean ± SD.

**FIGURE 8:**
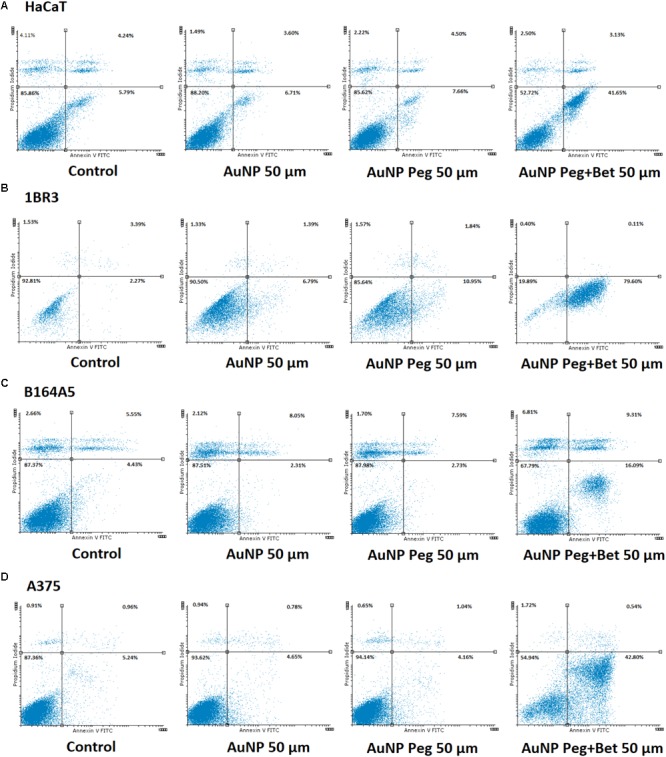
Representative dotplots of apoptotic cells stained with Annexin V-FITC/PI and analyzed by flow cytometry after 72 h treatment, grouped as follows: **(A)** HaCat: Control, AuNP 50 μM, AuNP Peg 50 μM, AuNP Peg + Bet 50 μM; **(B)** 1BR3: Control, AuNP 50 μM, AuNP Peg 50 μM, AuNP Peg + Bet 50 μM; **(C)** B164A5: Control, AuNP 50 μM, AuNP Peg 50 μM, AuNP Peg + Bet 50 μM; and **(D)** A375: Control, AuNP 50 μM, AuNP Peg 50 μM, AuNP Peg + Bet 50 μM. Selection of the dotplots was based on the significant pro-apoptotic effects induced by test compounds at 50 μM.

**Figure 7** represents the percentage of early apoptotic cells of HaCaT, 1BR3, B164A5 and A375 cell lines after 72 h of incubation with the tested substances while **Figure 8** exhibits representative dot plots for the flow cytometric Annexin V/PI analysis of each cell line after 72 h of treatment. One can notice that AuNP and AuNP Peg had no semnificative effect on cell apoptosis in case of B164A5 and A375 melanoma cell lines, while in case of HaCaT and 1BR3 cells a slight pro-apoptotic effect was observed for AuNP Peg. However, when betulin was included in nanoparticles, a strong dose-dependent apoptotic effect was observed for all cell lines.

## Discussion

The use of gold nanoparticles as carriers for betulinic acid was previously reported as effective in the management of cervical cancer (Jiang and Wang, 2016), the drug transport into the cancer cells increasing due to nanoparticle endocytosis. In the current paper we accomplished the synthesis of betulin loaded gold nanoparticles based on the hypothesis that gold conjugation will improve drug bioavailability and, consequently, its anticancer properties.

The synthesis of the gold nanoparticles was conducted by the slightly modified Turkevich method which involved the use of sodium citrate to reduce the chloroauric acid to metallic gold; the method allows the formation of almost monodisperse spherical gold nanoparticles with diameters smaller than 50 nm (Stiufiuc et al., 2013). The selection of the synthesis method was determined by the fact that unlike other more complicated shapes, spherical nanoparticles are easily synthesized using weakly bonded (i.e., hydrogen bonds) stabilizing ligands which enable ligand exchange; therefore, spherical gold nanoparticles are suited as drug delivery agents (Dreaden et al., 2012). In addition, spherical nanoparticles show superior celullar uptake to nanorods (Arvizo et al., 2010). The resulting gold nanoparticles exhibit modified surfaces, displaying attached citrate moieties that also act as stabilizer against particle aggregation (Dobrowolska et al., 2015). The colloidal gold solution with a diameter ranging between 5 and 60 nm is stable without adding a supplementary stabilizer (Verma et al., 2014).

An important issue in nanoparticle administration is the intervention of the reticuloendothelial system (RES); previous reports mentioned the passive targeting of RES by gold nanoparticles ranging between 10 nm and 250 nm up to 46% (Watkins et al., 2015). For this reason, PEG coating can be used to increase biocompatibility and circulation time as well (Watkins et al., 2015) due to the tight association of the ethylene glycol units with water molecules, the resulting hydrating layer preventing subsequent clearance (Blanco et al., 2015). We used as capping agent a thiolated PEG with medium molecular weight (3500 Da) based on a 2013 study which revealed that, although, high molecular weight PEG are more efficient stabilizers than low molecular weight PEG, the latter produces higher saturated capping density which allows a more efficient functionalization of gold nanoparticles (Wang et al., 2013). The thiolated PEG used in our experiments also presented a carboxyl termination that induced high stability, biocompatibility and excellent binding properties (Park et al., 2013).

The formation of the stabilized gold nanoparticles was monitored at regular time interval throughout the synthesis by UV-Vis spectroscopy. The presence of the absorption maximum in the range of 500–550 nm clearly indicates the formation of the gold nanoparticles as reflected by the color change of the gold colloidal solution from colorless to ruby-red (Singh et al., 2014). There is a direct correlation between the wavelength and the particle size and between the width of the peak and the particle size distribution as well (Huang and El-Sayed, 2010; Dhamecha et al., 2016); therefore we can state that the final gold colloidal solution contains nanoparticles with a narrow size distribution, results confirmed by the other methods applied in the present work.

The FTIR combined with ICP-MS analysis confirmed the presence of gold, PEG and betulin in the respective samples (**Figure 1B** and **Table 1**); all the absorption bands were attributed to vibrational modes according to the literature (Schrader, 1995; Vonnemann et al., 2014). The results are in agreement with the other analytical methods thus providing evidence for the formation of nanoparticles carrying a stabilizer.

The gold nanoparticles were characterized by employing consecrated analytical techniques such as TEM imaging and DLS measurements. The images obtained as a result of TEM analysis do not capture the organic exterior layers at the gold surface; TEM provides accurate information only regarding the metallic core. The average dimension of the gold nanoparticles is located around values of 14–15 nm as revealed by TEM analysis. Following DLS measurements one can notice a larger diameter of nanoparticles, around 50 nm in case of naked AuNP or betulin conjugated-AuNP due to an electric dipole at the metallic surface as a result of water dispersion (Spaas et al., 2016). When PEG was used as coating agent, TEM analysis indicated the same dimension of 14–15 nm for the metallic core while the DLS measurement provided an average dimension of 70 nm presumably attributed to the hydrated chains of PEG covalently bonded to the surface. The nanoparticles’ size plays an important role in tumor uptake and tissue penetration; Huo et el. (2013) reported that in spite of the limitations of drug delivery deep into the tumor tissue, imposed by tumor microenvironment, nanoparticles with an average diameter of 50 nm were superior to larger ones (100 nm diameter) in terms of tissue permeability. Moreover, the passive targeting of solid tumors was additionally facilitated by the enhanced permeation and retention effect (EPR) (Huo et al., 2013); nanoparticles with small sizes (below 50 nm) show a wider biodistribution into the human body as compared with larger ones (i.e., 200 nm) (Garrido et al., 2015). These nanoparticles are able to penetrate the blood brain barrier and have the longest half-life when coated with PEG (Arvizo et al., 2010). In light of these findings the average dimensions of 15 and 50 nm for naked AuNP and AuNP Peg, respectively, obtained during the course of our experiments, are appropriate in terms of cancer cell penetration for future therapeutic approaches.

The zeta potential provides an electrostatic stabilization factor of suspended nanoparticles and characterizes their surface charge, highly depending on other factors such as solvent, pH, functional groups (Zhang et al., 2014). Literature reports that a zeta potential value ranging from -30 mV to 30 mV is specific for a stable dispersed system due to rejection between nanoparticles. The values reported in our experiments are consistent with the literature specification, all dispersed systems revealing physical stability during storage, in particular in the presence of an additional stabilizer such as thiol-PEG-COOH (Zhang et al., 2014). The increase of the zeta potential values (**Table 2**) for the PEGylated AuNP reflect the capping of the metallic surface with PEG-SH (through covalent Au-S bonding) and with betulin, presumably through electrostatic or hydrogen bonds (formed between the carboxyl termination of the PEG-SH and the hydroxyl group of betulin). The attachment of the drug molecule through non-covalent interactions such as electrostatic or hydrogen bonds provides nanoparticles’ stability and the ability to release the conjugated drug in acidic environments (i.e., cancer cell) as a result of carboxyl ionization (Madhusudhan et al., 2014).

The gold nanoparticles (AuNP, AuNP Peg with or without betulin load) synthesized in the present study were assessed in terms of cytotoxicity and induction of apoptosis in non-malignant (human keratinocytes – HaCaT and fibroblasts – 1BR3) and cancer cells (human – A375 and murine – B164A5 melanoma). Previous studies demonstrated the effectiveness of gold nanoparticles in damaging cancer cells (Xu et al., 2012; Li et al., 2017; Lu et al., 2017; Lopez-Chaves et al., 2018) via several pathways, including: cell death, stimulation of pro-apoptotic protein (Bax) expression, inhibition of tumor cells migration and motility (Lu et al., 2017), suppression of epithelial-to-mesenchymal transition (Li et al., 2017) and oxidative reactive species (ROS) production after short-exposure time (Lopez-Chaves et al., 2018). It is considered that the appropriate size of gold nanoparticles for tumor therapy in humans is 50 nm, whereas the size required for the ones employed as diagnostic tools is smaller -20 nm (Agunloye et al., 2017). In a recent study, Lu and co-workers showed that AuNPs of different sizes (1–3; 3–5, and 10–15 nm) exerted cytotoxic effects on a murine melanoma cell line B16F10 in a dose-dependent manner, the nanoparticles with the smallest size being the most cytotoxic. Moreover, in the same study, it was observed that the smallest AuNPs (1–3 nm) were highly toxic against non-malignant human keratinocytes (HaCaT) cells after 72 h treatment as compared to the bigger ones (10–15 nm) which were barely cytotoxic (Lu et al., 2017). Our results are somehow in agreement with the aforementioned data, since the AuNPs (14–15 nm – dimensions of the metallic core) induced a time and dose-dependent cytotoxicity in human (A375) and murine (B164A5) melanoma cells, the most significant effect being observed at the highest concentration tested – 50 μM (**Figure 5**). In the case of AgNP PEG coated, there were no significant changes regarding the viability of melanoma cells. Stimulation of HaCaT cells with both AuNP and AuNP PEG coated was associated with a lack of toxicity even at the highest doses tested (**Figure 6**), indicating a safe profile, results that are consistent with the data from the literature. Assessment of the cytotoxic effects of AuNP and AuNP PEG coated on human fibroblasts – 1BR3 led to a different result concerning AuNP stimulation which exerted a dose and time-dependent toxic effect (**Figure 6**). Our results proved that AuNP PEG coated could be considered safe for human fibroblasts, since no significant toxic effect was recorded (**Figure 6**).

The efficacy of betulin as an anticancer agent has been previously reported by our group in various tumor cell lines, such as skin epidermoid carcinoma (A431), cervix adenocarcinoma (HeLa), breast carcinoma (MCF7) and murine melanoma (B164A5) (Alakurtti et al., 2006; Dehelean et al., 2012; Soica C. M. et al., 2012). Furthermore, there are studies indicating that betulin decreases melanoma cells viability and induces apoptosis (G-361 human melanoma cells, (Orchel et al., 2014); B164A5 and B16F0 murine melanoma cells, (Pfarr et al., 2014, 2015)). One of the main disadvantages of this compound is represented by its poor water solubility that diminishes its bioavailability (Dehelean et al., 2013). In an attempt to improve the solubility, our group has obtained different betulin formulations, namely nanoemulsions (Dehelean et al., 2013) and cyclodextrin complexes (Soica C. et al., 2012) that proved an enhanced therapeutic activity. Also, the literature contains a few reports concerning the synthesis of betulin nanoformulations such as carbon nanotubes or polymeric vectors (**Table 4**) with antiproliferative activity. One can notice that, despite the intrinsic antiproliferative activity of pure betulin, its nanoformulations provide improved biological effects either *in vivo* (nanoemulsions) or *in vitro* (cyclodextrin complexes, carbon nanotubes, polymeric vectors).

**Table 4:** Biological activities of different betulin formulations.

Formulation	*In vitro* cytotoxic/antiproliferative activity	*In vivo* antitumor activity	Reference
Betulin	No significant cytotoxic effect on J774A.1 macrophage cell line (IC_50_ value is 211.05 ± 7.14 μg/ml)		Saudagar and Dubey, 2014
Betulin	Decreased viability of B164A5 murine melanoma cells (52% ± 0.09% viable cells).		Soica C. et al., 2012
Betulin	Cytotoxic effect on cervix carcinoma HeLa cells, hepatoma HepG2 cells, lung adenocarcinoma A549 cells, and breast cancer MCF-7 cells (IC_50_ values ranging from 10 to 15 μg/mL); Moderate anticancer activity in hepatoma SK-HEP-1 cells, prostate carcinoma PC-3 cells, and lung carcinoma NCI-H460 cells (IC_50_ values ranging from 20 to 60 μg/mL)		Li et al., 2010
Betulin	Antiproliferative effect on Neuroblastoma SK-N-AS cells, glioma C6 cells, rhabdomyosarcoma-medulloblastoma TE671 cells, colon adenocarcinoma HT-29 cells, breast carcinoma T47D cells, thyroid carcinoma FTC238 cells, lung carcinoma A549 cells, multiple myeloma RPMI8226 cells, T cell leukemia Jurkat 1E.6 cells, ovarian carcinoma HPOC cells, cervical carcinoma HPCC cells and glioblastoma multiforme HPGBM cells (IC_50_ values ranging from 2.5 to 10.3 μM)		Rzeski et al., 2009
Betulin	Antiproliferative effect on A431 (skin epidermoid carcinoma), HeLa (cervix adenocarcinoma) and MCF7 (breast adenocarcinoma) (IC_50_ values are 6.76, 6.67, and 8.32 μM)	Antiangiogenic effect by reducing the newly formed capillaries and affecting the normal function of endothelial cells.	Dehelean et al., 2012
Betulin nanoemulsion		BetNE reduced skin tumor apparition and promotion in the two-stage skin carcinoma (DMBA and TPA application); Increased basal and ADP-stimulated respiration in isolated liver mitochondria from treated mice. In CAM assay – BetNE decreased the density of capillaries vs. NE alone.	Dehelean et al., 2013
Betulin complexes with cyclodextrins	Decreased B164A5 murine melanoma cells viability (62% ± 0.25% viable cells); Increased number of apoptotic cells (5.38% vs. control 4.32%) and dead cells (10.51% vs. control 5.39%)	Decrease in tumor volume in C57BL/6J mice with B164A5 cells inoculation; Reduced expression of S100 and VEGF in Bet:GCDG 1:1 treated mice	Soica C. et al., 2012
Carbon nanotubes loaded with betulin	No significant cytotoxic effect on J774A.1 macrophage cell line (IC_50_ value is 72.63 ± 6.14 μg/ml.)		Saudagar and Dubey, 2014
Poly-D,L-lactide nanovectors (PLA NVs) with encapsulated betulin	Anticancerous activity against cervical carcinoma SiHa cells (up to 80% cell inhibition following 72 h incubation)		Yadav et al., 2016
Gold nanoparticles conjugated with betulin	Antiproliferative activity against B164A5 (30–40% viable cells), A375 (30–35% viable cells)		Current paper

Another method to increase betulin solubility in aqueous solutions and to augment its bioavailability was proposed in the current study, by manufacturing betulin-conjugated gold nanoparticles, compounds that to the best of our knowledge were prepared for the first time and represent one of the novelty elements of our study. Furthermore, we consider this type of nanoformulation to be superior, in terms of drug loading/release, to other formulations containing triterpenes such as drug-cyclodextrin complexes. In a previous work we reported the formulation of a betulin complex with a cyclodextrin derivative containing hydrophobic substituents that showed a very high stability constant, which impeded drug release at the targeted location (Soica C. et al., 2012). Considering their large surface area available for molecule binding and relatively weak drug-nanoparticle interactions (Huo et al., 2013), spherical gold nanoparticles could provide improved quantitative drug release at the tumor site.

Our data showed that the nanoparticles loaded with betulin (both naked and coated with PEG) exhibited potent cytotoxic effects against melanoma cells in a dose and time-dependent manner, the human melanoma cells being more sensitive to their toxic effects. Another very important finding was that these nanoparticles showed significant cytotoxicity compared to betulin (when tested under the same experimental conditions in terms of concentration and time points of incubation). The most significant cytotoxic effects against A375 were recorded at 48 h post-stimulation, whereas in the case of B164A5 cells the toxicity was more prominent after 72 h treatment (**Figure 5**). In addition, the current study furnishes important information regarding the impact of betulin loaded gold nanoparticles on human keratinocytes and fibroblasts, as follows: HaCaT cells viability was affected in a dose and time-dependent manner after both type of nanoparticles, but did not decrease under 50%, albeit 1BR3 proved to be very sensitive to these nanoparticles, the cytotoxic effect being dose-dependent and visible after 24 h (**Figure 6**). The results are in line with the ones obtained from Annexin V/PI analysis which revealed that betulin loaded nanoparticles induced a potent apoptotic effect for all the cell lines.

## Conclusion

Present study reported the synthesis, characterization and biological activity assessment of various surface modified betulin conjugated gold nanoparticles. The compounds synthesized via reduction method using trisodium citrate as reducing agent and chloroauric acid as gold precurssor, presented a stable size that was confirmed by various techniques. Average dimension for all types of synthesized gold nanoparticles was around values of 14–15 nm (measurement related only to the metallic core regardless of the modified surface). On the other hand, according to DLS measurements, larger diameters of nanoparticles were obtained, ranging from 50 to 70 nm (dependent on the type of nanoparticle synthesized). The differences between final products are dependent on different causality factors responsible for the size gap using the different methods, the presence of an electric dipole at the metallic surface, as a result of water dispersion (for naked AuNPs) or the presence of a modified surface with large molecules (PEG-SH). The *in vitro* evaluation of the tested samples indicated that betulin-conjugated gold nanoparticles determined a cytotoxic and apoptotic effect in a dose-dependent manner on all cell lines and is dependent also on exposure time. Naked AuNP or coated with Peg modified only slightly cells viability.

Betulin conjugated gold nanoparticles can be considered a new therapeutic strategy for patients with melanoma. Further *in vivo* studies will be performed in order to establish the effect in experimental models of skin cancer.

## Author Contributions

MM: effectuated the synthesis of the gold nanoparticles. RG and VS: performed the physico-chemical characterization, analyzed the data, and drafted the work. IZP, CO, DC, and CF: performed the *in vitro* tests, analyzed and interpretation of the data acquired, and drafted the work. C-VM: performed TEM assay, acquired and analyzed the data, and drafted the work. MM and IZP prepared the manuscript for submission. CŞ, RP, and CD: elaborated the final version of the manuscript, corrected the language, and critically revised the work. MS and AT evaluated the scientific work.

## Conflict of Interest Statement

The authors declare that the research was conducted in the absence of any commercial or financial relationships that could be construed as a potential conflict of interest.
